# Development of the “SMA NNE,” a short neonatal neurological examination for newborns with spinal muscular atrophy

**DOI:** 10.1007/s00431-025-06382-4

**Published:** 2025-08-19

**Authors:** Eugenio Mercuri, Giorgia Coratti, Costanza Cutrona, Roberto De Sanctis, Giulia Stanca, Gianpaolo Cicala, Marianna Villa, Anna Capasso, Chiara Arpaia, Martina Sbarbati, Kristin Krosschell, Domenico Romeo, Beatrice Berti, Richard Finkel, Marika Pane

**Affiliations:** 1 Child Neurology and Psychiatry Unit, Fondazione Policlinico Universitario Agostino Gemelli IRCCS, Rome, Italy; 2 Centro Clinico Nemo Pediatrico, Fondazione Policlinico Universitario Agostino Gemelli IRCCS, Rome, Italy; 3 Pediatric Neurology, Catholic University, Rome, Italy; 4 Department of Pediatric Neurorehabilitation, IRCCS Santa Lucia Foundation, Rome, Italy; 5 Northwestern University Feinberg School of Medicine, Chicago, IL USA; 6 Center for Experimental Neurotherapeutics, Department of Pediatric Medicine, St. Jude Children’s Research Hospital, Memphis, TN USA

**Keywords:** Neonatal, Newborn screening, Spinal muscular atrophy, Neonatal neurological examination

## Abstract

The advent of newborn screening for spinal muscular atrophy (SMA) has highlighted the need for easy, quick, clinical tools to be used in infants with SMA identified in the neonatal period. We propose a new short module developed using items from the Hammersmith Neonatal Neurological Examination (HNNE) and from a module developed for floppy infants, both previously used in newborns with SMA. The new module was developed by identifying and selecting the items that were more often found to have abnormal findings in SMA newborns. The proforma was designed by selecting 13 items and converting them into a format that would facilitate the interpretation of the results. The cohort included 25 infants. Based on HNNE and add-on module results, 13 were classified as asymptomatic, 5 as pauci-symptomatic, and 7 as symptomatic. All symptomatic infants showed abnormalities in posture, tone, tremors, and reflexes, along with reduced antigravity movements and abnormal breathing. Among pauci-symptomatic infants, all had abnormal reflexes, three also had tremors, and one showed mild hypotonia. There was excellent inter-observer reliability assessed using intra-class correlation coefficients with 95% confidence intervals (0.947) and full concordance with the original full-length forms.

*Conclusion*: Our findings support the use of the short form in clinical practice, especially when time or resources are limited. It can be used in multiple occasions, allowing to detect the onset of possible signs in asymptomatic infants and to follow their progression.

**Table:** 

**What is Known:** *• Newborns with SMA identified by neonatal screening may show clinical signs at diagnosis.* *• These signs may be subtle and easily missed on standard neonatal examination.* **What is New:** *• The new form includes a short examination that can reliably detect initial signs of SMA.* *• The new examination can be used to detect the onset and the progression of clinical signs.*

**What is Known:**

*• Newborns with SMA identified by neonatal screening may show clinical signs at diagnosis.*

*• These signs may be subtle and easily missed on standard neonatal examination.*

**What is New:**

*• The new form includes a short examination that can reliably detect initial signs of SMA.*

*• The new examination can be used to detect the onset and the progression of clinical signs.*

## Introduction

Spinal muscular atrophy (SMA) is an autosomal recessive neuromuscular disease caused by mutations in the survival motor neuron 1 (*SMN1*) gene, with degeneration of spinal motor neurons resulting in generalized muscle hypotonia, weakness, and atrophy [1]. Before the advent of disease-modifying therapies (DMTs), infants with signs of SMA at birth or in the neonatal period were considered to have a poorer prognosis, with a very reduced survival [2, 3]. The advent of disease-modifying therapies has dramatically changed survival and disease course, even in the cases with neonatal onset, with better results in the newborns that can be identified at birth using newborn screening (NBS) [1]. The early identification of newborns with SMA by NBS has however highlighted the need to have appropriate tools to be used in clinical practice at the time of diagnosis and in the follow-up. Until relatively recently, newborns identified via NBS were broadly subdivided into “presymptomatic” and “symptomatic,” including in the second group those who at birth or between birth and time of diagnosis had developed obvious clinical signs suggestive of SMA [4]. The infants labeled as symptomatic were those with evidence of weakness, hypotonia, diaphragmatic breathing, and other clinical signs that, because of the early onset, were compatible with a classification of SMA type 1.1 [5, 6] or 1a [2, 3] or, in the most severe cases with contractures and evidence of prenatal findings, as type 0 [7]. The review of the first studies and trials reporting the results of NBS has revealed that a number of “presymptomatic” patients, even if not showing the full clinical picture of SMA, still had subtle neurological signs consistent with early presentation of the disease [8]. Finkel and Benatar proposed a conceptual framework to better characterize and identify these infants [4], describing a “prodromal disease” to include the infants in whom there is evidence of subtle emergence of symptoms. These infants have also been reported as “pauci-symptomatic.” This presentation was consistent with evidence from the review of the infants included in the first clinical trial in “presymptomatic” patients, NURTURE (NCT02386553) [9], suggesting that a number of them already had absent or weak reflexes at the time of enrollment that would have allowed to classify them as pauci-symptomatic. Real-world data from infants identified by NBS have confirmed similar findings [8, 10, 11]. These infants also often have lower compound motor action potential (CMAP) values and increased neurofilaments, and it has been suggested that they should be assessed using an integrated approach including a standardized clinical assessment [8, 10, 11]. We recently reported our experience on a small cohort of newborns identified via NBS assessed using the Hammersmith Neonatal Neurological examination (HNNE), a structured neonatal neurological assessment [12], and an add-on module specifically designed to identify clinical signs such as weakness, contractures, or breathing patterns suggestive of neuromuscular disorders in floppy newborns and infants [13].

The combined use of the two assessments allowed us to identify newborns in all three of the nosological entities suggested by Finkel and Benatar [4], including 3 of the 17 infants who presented with minimal signs, consistent with the prodromic phase and labeled as pauci-symptomatic. The signs that were found to be present, even in the absence of the unequivocal hypotonia, were weak/absent reflexes and tongue fasciculations [8]. A 24-month follow-up of the same cohort showed that the initial clinical neurological signs were related to the timing of developmental milestone acquisition and to the overall motor outcome [14]. More specifically, pauci-symptomatic infants achieved the same milestones as the asymptomatic ones but with delay.

These results were promising, suggesting that the combined use of HNNE and the add-on module could reliably allow the classification of newborns identified via NBS in the three main nosological categories (symptomatic, pauci-symptomatic/prodromal, and asymptomatic). Both the HNNE and the add-on module have been designed to be self-explanatory and do not require specific high-level training, with the HNNE having been used for decades in clinical and research setting. Nevertheless, many clinicians, especially those who have less experience with neonates, are less keen to perform a full structured neonatal neurological assessment that also includes items that are not suitable to specifically identify possible clinical signs of SMA.

Based on our experience of adapting the full HNNE to create a shorter version for screening newborns at risk for other conditions, such as prematurity [15], or for use in low-resource settings [16], we developed the SMA NNE (Spinal Muscular Atrophy Neonatal Neurological Examination), a new short proforma specifically designed for infants identified by NBS for SMA. This paper reports the development of the new shortened examination, starting from the analysis on the available data using both the HNNE and the add-on module in both low-risk newborns and in those with SMA identified via NBS, in order to identify the items that could more specifically identify both the fully symptomatic newborns and the pauci-symptomatic ones.

## Materials and methods

The study included infants identified through NBS for SMA and enrolled after September 2019 as part of a larger multidisciplinary research project aimed at identifying early biomarkers as part of a research project assessing clinical and laboratory findings in SMA in a multidisciplinary network (PNRR-MR1-2022–12376937).

Included infants had a confirmed diagnosis of SMA before 2 weeks of age, with prospectively collected clinical and laboratory data available for analysis.

As part of our protocol, infants were assessed using the HNNE, a structured neonatal neurological examination [12] and an add-on module specifically developed for identifying neuromuscular disorders in floppy infants [13]. Gestational age, age at diagnosis, sex, and SMN2 copy number were also noted at the time of enrollment.

All the assessments were performed by experienced pediatric neurologists or physiotherapists with experience both in neuromuscular disorders and neonatal neurology.

This study was performed in line with the principles of the Declaration of Helsinki. Approval was granted by the Ethics Committee of the Catholic University of Rome (prot. N. 003,475/21).

Written informed consent was obtained in all cases.

The analysis of the data followed different steps:

- *Identification and selection of items more often with abnormal results in SMA newborns and development of a shorter combined form using the selected items*.
- Preliminary data from the use of these two tools had already shown that the assessments can allow to identify both pauci-symptomatic and fully symptomatic patients among those identified via NBS [8]. Data from the original cohort and from additional infants identified after the first study were analyzed identifying the items that were found to be abnormal on the two forms used in the SMA infants identified by NBS [12, 17]*.*
- *Modification of the proforma using the screener format.*
- One of the difficulties encountered by clinicians with less experience in neonatal neurology is that in the original full version for each item the proforma includes a number of options that reflect the possible variability of findings which are optimal at different gestational ages. These are reported as a continuum, and it is therefore difficult for less-experienced clinicians to immediately understand if what is observed and reported on the form should be considered normal or abnormal, especially in the context of gestational age. In this study we aimed to apply the same criteria used for developing shorter screeners for both full term and preterm infants at term age, using a new format that allows an immediate interpretation of the results. Each item was redesigned according to the frequency distribution of findings in data collected in low-risk full-term and premature newborns examined at term age. Each of the items is set out in three columns. The central column is designed to report the spectrum of neurologic findings and scores seen in the typically developing population within the reference range (90%) for full-term and preterm infants [12, 18] at term age, while the findings in the two lateral columns report what should be considered suboptimal or as “warning signs” for SMA.

The new proforma was tested in a cohort of seven newborns to establish interobserver reliability.

- *Validation of the short proforma against the original full forms*.
- To establish whether the SMA NNE was able to detect the findings identified as abnormal in the full examination, we transposed all the individual findings from the full examinations into the new short module. This was possible as all the findings for the items contained in the short module were included in the full examination.

### Statistical analysis

Descriptive statistics summarized the demographic and clinical characteristics of the study population. Continuous variables were presented as means with standard deviations (SD) or medians with ranges (min–max), while categorical variables were reported as frequencies and percentages. Given the rarity of the disease, the sample size was not predetermined and was determined by the number of newborns identified through screening during the study period. Inter-observer reliability was evaluated using intra-class correlation coefficients (ICC) with 95% confidence intervals (CI), based on a two-way random effect, single-measures analysis of variance model. Concordance between the short proforma and the full-length forms was assessed using Cohen’s kappa coefficient.

## Results

The cohort included the 17 previously reported [8] infants and 8 additional ones. Twenty-three of the 25 (14 females and 11 males) were born at term age (median: 39 weeks, range: 38–41 weeks), one at 37 week-, and one at 31-week gestational age. Fourteen of the 25 had 2 *SMN2* copies, 4 had 3 copies, 4 had 4 copies, and 2 more than 4 copies. In all the examination was performed in the first week after birth soon after the identification by NBS.

Thirteen had completely age-appropriate findings on all the items on both HNNE and add-on module and were defined as asymptomatic, five had subtle signs on one or more items and were defined as pauci-symptomatic, and seven had multiple abnormal results and were defined as fully symptomatic. Among the symptomatic, 1 had type 0 with 1 *SMN2* copy. One was asymptomatic on the first assessment but became symptomatic by the time he had confirmation 3 days later. Table 1 summarizes demographic and clinical data from the cohort.

**Table 1:** Demographic and clinical data from the cohort subdivided by symptoms

	Asymptomatic (*N* = 13)	Pauci-symptomatic (*N* = 5)	Symptomatic (*N* = 7)	Overall (*N* = 25)
SMN2 copy number				
1	0 (0%)	0 (0%)	1 (14.3%)	1 (4.0%)
2	4 (30.8%)	4 (80.0%)	6 (85.7%)	14 (56.0%)
3	3 (23.1%)	1 (20.0%)	0 (0%)	4 (16.0%)
4 +	6 (46.2%)	0 (0%)	0 (0%)	6 (24.0%)
Sex assigned at birth				
Female	8 (61.5%)	1 (20.0%)	2 (28.6%)	11 (44.0%)
Male	5 (38.5%)	4 (80.0%)	5 (71.4%)	14 (56.0%)
Gestational age				
Mean (SD)	39.0 (0.91)	39.6 (1.34)	38.7 (3.45)	39.0 (1.95)
Median [min, max]	39.0 [37.0, 40.0]	39.0 [38.0, 41.0]	40.0 [31.0, 41.0]	39.0 [31.0, 41.0]
Age at assessment				
Mean (SD)	0.06 (0.067)	0.26 (0.43)	0.03 (0.01)	0.09 (0.20)
Median [min, max]	0.04 [0.02, 0.28]	0.06 [0.04, 1.03]	0.03 [0.02, 0.04]	0.04 [0.02, 1.03]

*Identification and selection of items more often presenting with abnormal results in SMA newborns and development of a shorter combined form using the selected items*.

Among the symptomatic patients (*n* = 7), all exhibited abnormal scores in posture, tone, tremors, and tendon reflexes on the HNNE (100%).

Additionally, all demonstrated reduced antigravity movements and abnormal breathing patterns on the add-on module (100%). Sucking and swallowing abnormalities were present in a subset of symptomatic patients (*n* = 2, 24%). The patient classified as type 0 (*n* = 1) also presented with congenital contractures.

In the pauci-symptomatic patients (*n* = 5), all had abnormal reflexes (100%), three also had tremors, and one also presented with some degree of hypotonia. Based on the pattern and frequency of clinical findings, the shorter integrated proforma includes 13 items: the item assessing posture, one item assessing upper limb tone, one assessing lower limb tone, and two items assessing head control in flexion and extension (head lag and ventral suspension) from the HNNE, together with the item assessing tremors. The items selected from the add-on module were tendon reflexes, antigravity movements (both upper and lower limbs), respiratory pattern, sucking, and swallowing. The two original items on contractures were collapsed into one, as neonatal contractures in SMA are mainly relevant for type 0. Other items, such as facial weakness, that are important for other neuromuscular disorders, but not for SMA, were also excluded.

### Modification of the proforma using the screener format

**Fig. 1:**
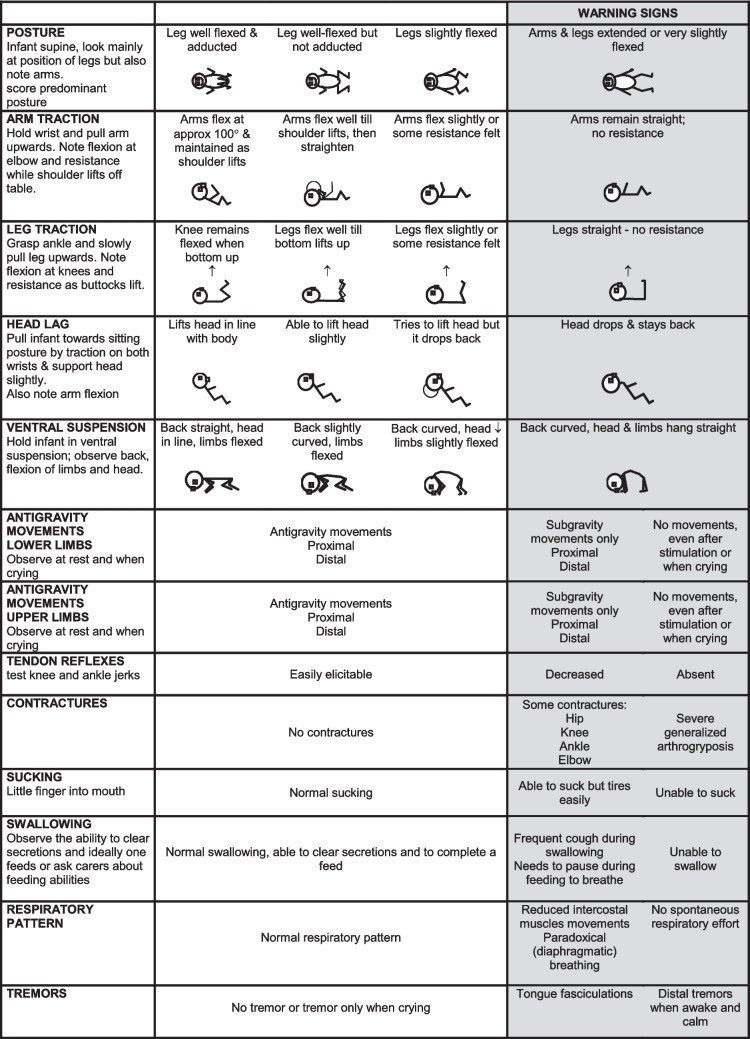
Final form of the Spinal Muscular Atrophy Neurological Neonatal Examination (SMA NNE)

The 13 items included in the final integrated short proforma were converted into the format previously used for the short routine examination for both full-term and preterm newborns, reporting optimal results in the first column (found in 90% of the reference data according to the frequency distribution of findings in data collected in low-risk full-term and premature newborns examined at term age). The second and third columns included the findings observed in less than 10%. The proforma was further simplified deleting, for each item, drawings, and findings that were specific for central nervous system (CNS) involvement and considered not appropriate for our purpose (Fig. 1). The assessment of interobserver reliability among two examiners was 0.947 (*n* = 7, *p* < 0.001).

### Validation of the shorter proforma against the original full forms

To validate the shorter proforma against the original full-length forms, patient classifications were compared across both instruments. All 13 patients classified as asymptomatic by the full examination had scores exclusively in the first column of the SMA NNE on all items, with no indication of warning signs. Conversely, the five pauci-symptomatic and seven symptomatic patients demonstrated abnormal findings, with all relevant scores falling outside the first column in the SMA NNE, corresponding to warning signs. A Cohen’s kappa coefficient was calculated to assess concordance between classifications derived from the short proforma and the full-length forms. The kappa value was 1.0 (95% CI: 1.0–1.0).

## Discussion

We report the development of an easy, quick clinical tool to be used in clinical practice for the newborns with SMA identified via NBS. This new module was developed after using the HNNE, a structured neonatal neurological assessment, together with an add-on module developed for the differential diagnosis for floppy infants. The HNNE has been widely used worldwide in both preterm and full-term infants since the 1970 s and has been extensively used to follow normal maturation or as part of integrated approaches showing excellent correlation with brain imaging and neurophysiology in newborns with brain lesions [19]. As the HNNE had not been specifically developed to identify specific signs of neuromuscular disorders, a new tool, “the floppy module,” was recently developed as an add-on module for infants with hypotonia or at risk of neuromuscular disorders [13]. The two tools, in combination, have been used in newborns identified via NBS and have been able to identify both newborns with clinical signs of SMA and those with minimal signs that have been increasingly identified after the advent of clinical trials and NBS [8].

While both assessments are easy to perform and can be completed in less than 10 min with excellent reliability, there has been a request for a simpler and quicker tool, only including the most relevant and disease-specific items that could be easily used on multiple occasions after identification with NBS. The form reported in this paper focuses on a very restricted number of items. The selection of items was driven by the specificity of their observation in SMA infants, excluding many items that capture neurological aspects generally more suggestive of CNS involvement. The form only includes items that, historically and in our recent experience, were more often found to be abnormal in SMA, therefore also excluding other signs suggestive of peripheral involvement, such as facial weakness, that are less relevant in SMA [13, 20]. In order to improve the possibility to identify deviant or warning signs, the form was designed with a format similar to other previous modifications for screening purposes, i.e., not only reducing the number of items but also structuring the columns for each item so that findings that are less frequently observed in typically developing children can be easily identified. Since the new form retains the same drawings and instructions as the original version, we were able to directly map the findings previously collected with the HNNE and the add-on module in SMA patients onto the new format. In all cases all the findings that had been reported as abnormal in the original forms were in the last column which reflect findings observed in less than 10% of a reference population of low-risk full-term and preterm infants assessed at term-equivalent age. Similar results were also observed when piloting the new form in two newborns with SMA recently seen in our center.

The new module can be easily administered in less than 3 min. The ease of performing and scoring the examination in a few minutes with no need for extra equipment facilitates its repeated use on the multiple times when the infants are seen in the neonatal period. Even if there are differences in the timing of the appointments and in the setting among countries, and sometimes even among centers in the same countries [10, 21–24], the newborns identified via NBS are often seen several times within the first month. Our suggestion is to use the new module at the initial clinic visit when the family comes to perform confirmatory tests and to repeat it every time the family comes back in the neonatal period. This is equally important in both asymptomatic and symptomatic newborns. There has been increasing pressure to identify and treat newborns with SMA as soon as possible as a number of them may be asymptomatic at birth or at the time of diagnosis but develop overt signs and symptoms of SMA over the following days or weeks before they receive treatment. Capturing these changes on a structured form will help to justify the need to accelerate early treatment and, if gene therapy is the first choice but is not readily available, the use of other drugs for bridging. The new form is equally important in newborns who are already symptomatic at birth or at the time of diagnosis, allowing one to define the initial pattern of abnormal signs and the subsequent changes and monitor the onset and severity of the main signs typical of type 1 SMA, such as weakness, hypotonia, respiratory, and bulbar involvement.

In conclusion, our findings support the use of the new short integrated examination in clinical practice, especially when time or resources are limited as it can be reliably performed and can detect the warning signs that have been previously identified using the full HNNE and add-on module. Our suggestion is that, in the presence of warning signs, a more comprehensive assessment should be performed, also including neurophysiology [4] and biomarkers such as neurofilaments [25] that will provide additional information. The more comprehensive assessment should also include the Children’s Hospital of Philadelphia Infant Test Of Neuromuscular Disorders (CHOP INTEND), taking into account that some items are still developmentally immature in the neonatal period [26], and the full version of the HNNE and the add-on module. This is particularly important in the symptomatic newborns as the HNNE, also including a larger variety of items, including those assessing behavior, visual alertness, and irritability, may provide additional early information on cognitive and neurodevelopmental aspects for which there has been increasing attention [27, 28]. Further studies, prospectively using the new form in combination with other techniques in larger cohorts, will help to better define the diagnostic and prognostic value of this new tool.

## Data Availability

Data available on request.

## Abbreviation

*CNS*: Central nervous system
*CHOP INTEND*: Children’s Hospital of Philadelphia Infant Test of Neuromuscular Disorders
*CI*: Confidence intervals
*CMAP*: Compound motor action potential
*DMTs*: Disease modifying therapies
*HNNE*: Hammersmith Neonatal Neurological Examination
*ICC*: Intra-class correlation coefficients
*NBS*: Newborn screening
*SMA*: Spinal muscular atrophy
*SMA NNE *: Spinal Muscular Atrophy Neonatal Neurological Examination
*SMN1*: Survival motor neuron 1
*SMN2 *: Survival motor neuron 2
